# Supplementary material for: Bending skyrmion strings under two-dimensional thermal gradients
Source: Nat Commun. 2024 Jun 7;15:4860. doi: 10.1038/s41467-024-49288-9 (PMC11161597; doi:10.1038/s41467-024-49288-9)
Supplement: Supplementary file 1 — Supplementary Information [file 41467_2024_49288_MOESM1_ESM.pdf]

— Supplemental Material —

Bending Skyrmion Strings under Two-Dimensional Thermal Gradients

Kejing Ran,[1,2] Wancong Tan,[1] Xinyu Sun,[1] Yizhou Liu,[3] Robert M. Dalgliesh,[4] Nina-Juliane Steinke,[5] Gerrit van der Laan,[6] Sean Langridge,[4] Thorsten Hesjedal,[7] and Shilei Zhang[1,*]

[1]*School of Physical Science and Technology, ShanghaiTech University, Shanghai, China*

[2]*College of Physics & Center of Quantum Materials and Devices, Chongqing University, Chongqing 401331, China*

[3]*RIKEN Center for Emergent Matter Science (CEMS), Wako, Japan*

[4]*STFC, ISIS, Rutherford Appleton Laboratory, Didcot OX11 0QX, United Kingdom*

[5]*Institut Laue-Langevin, 38042 Grenoble, France*

[6]*Diamond Light Source, Didcot OX11 0DE, United Kingdom*

[7]*Department of Physics, Clarendon Laboratory, University of Oxford, Oxford OX1 3PU, United Kingdom*

(Dated: May 17, 2024)

* shilei.zhang@shanghaitech.edu.cn

S1. EXPERIMENTAL SETUP

Small angle neutron scattering (SANS) was performed on the LARMOR beamline at the ISIS neutron facility (Harwell, Oxfordshire, UK) using the time-of-flight technique. The coordinate system is defined in Fig.2 (b) in the main text, where the incident neutron beam is along x. Therefore, SANS primarily probes the q_y-q_z-plane in reciprocal space, while the q_x coverage is limited. The incident neutron wavelength ranges from 0.9 to 13.3 Å. By analyzing the scattered neutron spectrum, the q_x-component can be reconstructed. The cryostat is contained inside a superconducting vector magnet, which allows the magnetic field to be applied along any direction. The variable temperature insert (VTI, Oxford Instruments) is mounted inside the exchange-gas-based cryostat. Its rotational degree of freedom allows for performing rocking scans.

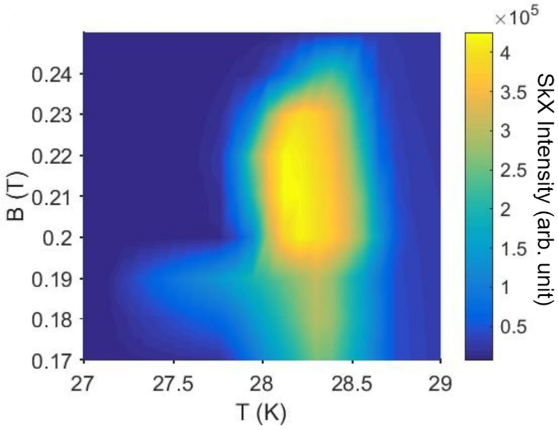

FIG. S1. Temperature vs magnetic field phase diagram mapped by integrating the SkX diffraction peak intensity.

In order to apply a wedge-shaped temperature gradient, a bespoke oxygen-free high thermal conductivity (OFHC) copper sample holder was made to accommodate our MnSi bulk sample. The holder is mechanically connected to the VTI stick, however, thermally isolated by a ceramic spacer. The MnSi single crystal [measuring $\sim 2.5 \times 15 \times 11\,\mathrm{mm}^3$; full-width at half maximum of the rocking curve of the (110) peak measured by Cu $K\alpha$ radiation is 0.0177°] was placed in a recessed part of the holder, and clamped down. As shown in Fig. 2(a,b) in the main text, a diode was used to heat the far-end of the holder, thereby avoiding Oersted fields. By applying a low current of 1-10 mA through the diode, the surface of the

lower end of the holder becomes the heat source, and heat flows through the sample holder towards the upper heat sink. Two Cernox sensors were mounted in order to monitor the real-time temperatures T_{A} and T_{B} and the lower front end (heat source) and the upper back end (heat sink), respectively, along with the SANS patterns.

The incident neutron beam with $\mathbf{k}_i$ is aligned along the [110] crystalline orientation of MnSi. This is achieved by balancing the rocking scan around the z-axis in the helical phase, in which four $\langle 111 \rangle$-locked helical peaks lie within the y-z-plane. Once $\mathbf{k}_i$ is optimized to be along [110] direction, no sample rotation was performed for the subsequent temperature-gradient-driven observations. Figure S1 shows the phase diagram mapped by the SANS peak intensity of the skyrmion crystal (SkX) phase. In order to study the 3D SkX rocking behavior under the 2D temperature gradient, we chose the magnetic field to be 0.22 T for stabilizing the SkX phase, at which the widest temperature range can be reached. As suggested by the phase diagram, due to the narrow temperature window of the SkX phase, the maximum difference between the hottest and coldest end cannot excess 0.5 K, otherwise part of the sample will enter the conical order.

S2. CALIBRATION OF THE TEMPERATURE GRADIENT INSIDE OF THE CRYSTAL

Here, we introduce our method that obtains the experimentally measured temperature gradient inside of the MnSi crystal. The measurement of the local temperature employed the sensitive response of the Pt resistivity to the temperature. For example, as shown in Fig. S2(b), the patterned microstructures of the Pt electrodes has a well-defined $\rho(T)$ profile. In particular, the slope of the resistivity curve is reasonably large within the temperature range between 27 K and 29 K. This offers an ideal calibration table for one to obtain the exact temperature value based on the resistivity reading.

In our case, we first polish the surface of the MnSi bulk crystal, on top of which an insulating SiO_2 layer with 5 nm thickness was deposited using magnetron sputtering. Subsequently, Pt electrodes (4 nm in thickness) were fabricated using thin film deposition and lithography techniques. We have fabricated four groups of the Pt electrodes (labeled as channel 1 to 4 as shown in Fig. S2), located at the four corners of the crystal, i.e., top (channel 1) and bottom (channel 2) on the front surface, as well as the top (channel 3) and

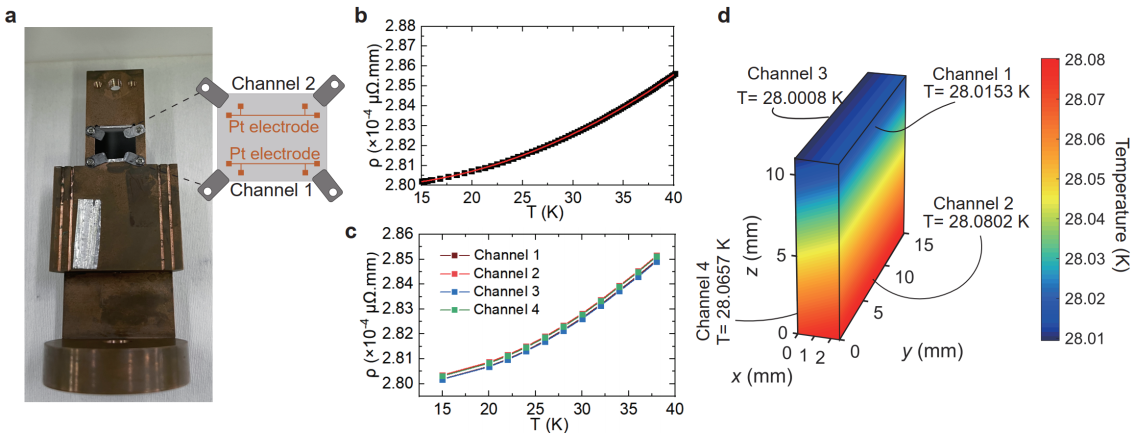

FIG. S2. (a) The configuration of the sample mounting mechanism, as well as the configuration of the Pt electrodes. (b) Typical Pt resistivity curve at the temperature range of interest. (c) The $\rho(T)$ curves for the four channels that are patterned on the four corners of the as-measured MnSi crystal. (d) Experientially calibrated temperature map inside of the MnSi crystal while the temperature gradient is on.

bottom (channel 4) on the back surface, respectively.

Next, we calibrate the $\rho(T)$ curves for all four channels in-situ without applying the diode current, i.e., the temperature distribution across the MnSi crystal is homogenous. Figure S2(c) shows the four $\rho(T)$ profiles measured simultaneously. Note that the four curves do not necessarily overlap due to the shape difference of the Pt electrodes, which is inevitable during the fabrication process. We would like to emphasis that despite of the fact that the four $\rho(T)$ curves exhibits minor difference, it does not affect the precision of the temperature reading. This is because each individual channel serves as its own calibration table.

At $\bar{T} = 28.05$ K, upon switching on the diode current, we immediately observed the resistivity change for all four channels. By converting the readout ρ-values into T-values, the temperature reading at the four corners are shown in Fig. S2(d). As our MnSi sample is a good single crystal, it is reasonable to assume a continuous thermal distribution throughout the entire bulk sample. We therefore obtain the experimentally measured temperature distribution in Fig. S2(d). It is thus clear that both $g_{\|}$ and $g_{\perp}$ well exist, validating the fact that we indeed applied 2D orthogonal temperature gradient by switching on the diode current. Moreover, we find minor difference between the experimentally probed temperature map and our finite-element simulation.

S3. MICROMAGNETIC SIMULATIONS

Micromagnetic simulations were performed using `Mumax3` [3]. The energy density, including the direct exchange (A), Dzyaloshinskii-Moriya interaction (DMI, D) and the Zeeman energy can be written as

$$w = A(\nabla \mathbf{m})^2 + D\mathbf{m} \cdot (\nabla \times \mathbf{m}) - M_\mathrm{S}\, \mathbf{m} \cdot \mathbf{B} \ , \tag{1}$$

where $\mathbf{m}$ is a unit vector along the local magnetization, and M_S, A, D, and $\mathbf{B}$ are the saturation magnetization, exchange stiffness constant, DMI constant, and external magnetic field, respectively. As we are considering a mm-sized bulk sample, we do not take demagnetization effects at the edges of the sample into account since they only contribute to a very small fraction to the overall observed dynamics.

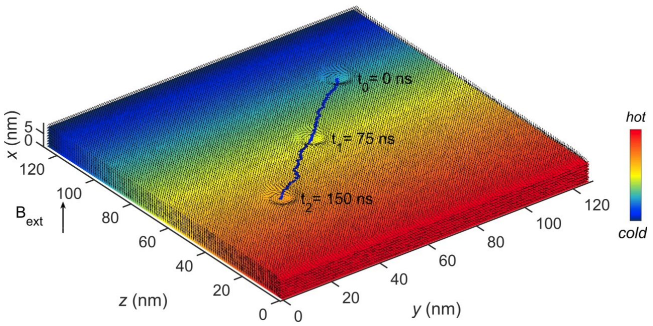

FIG. S3. Demonstration of the temperature dependence of the thermal-gradient-induced skyrmion motion using stochastic micromagnetic simulations. The colormap represents the local sample temperature. Blue line denotes the trajectory of the skyrmion for $\bar{T}/T_\mathrm{c} = 0.36$, $g_\parallel = 2.34$ K/nm.

To illustrate the temperature dependence of the skyrmion Hall effect more clearly, we first simulated the motion of single skyrmion under one-dimensional temperature gradients. A mesh size of $128 \times 128 \times 8$ and an elementary cell size of $1 \times 1 \times 1\,\mathrm{nm}^3$ were used. Open boundary conditions are applied in the z direction, and periodic boundary conditions in x and y. The Gilbert damping constant α is set to 0.1 for obtaining a larger stochastic field [4]. In the simulations, we set $M_\mathrm{S} = 1.04 \times 10^7$ A/m, $A = 3.547 \times 10^{-11}$ J/m, and $D = 3.71 \times 10^{-2}$ J/m^2. The advantage of using such relatively large parameters is to increase the Curie temperature T_C, so as to observe Θ_SH in a broadened temperature range $\bar{T}$.

As the initial state ($t_0 = 0\,\mathrm{s}$), a single skyrmion was chosen that was allowed to relax in

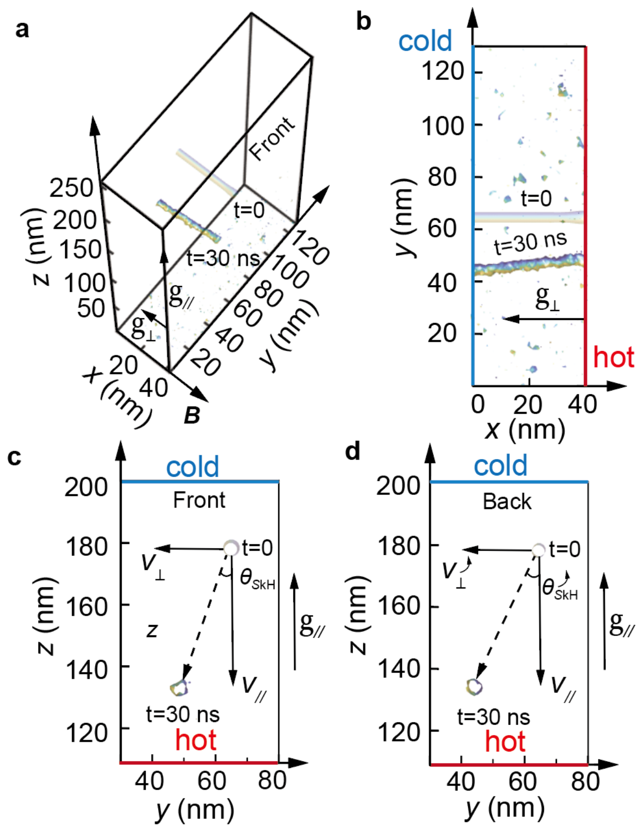

FIG. S4. (a) Micromagnetic simulation result of the 3D skyrmion string bending effect in a 2D thermal gradient, showing the skyrmion's string structures at two time stamps, i.e., $t = 0$ before the orthogonal temperature gradient is switched on; and $t = 30$ ns after $\mathbf{g}$ is switched on. (b) Side-view of (a), projecting the same information at xy-plane. The rotation of the string around z-axis (Ψ) due to $g_\perp$ can be clearly observed. (c,d) Skyrmion trajectories in the front and back yz-planes respectively.

the simulation. A 1D temperature gradient of $g_\parallel = 2.34$ K/nm was subsequently applied, driving the skyrmion towards the hot end, further exhibiting a deviation from the straight path along the direction of the gradient, i.e., a skyrmion Hall angle, as shown in Fig. S3. The temperature map is overlaid in the figure. We then track the skyrmion trajectory by varying $\bar{T}$, which clearly reveal the temperature dependence of the Hall angle Θ_{SkH}.

Figure S4 shows micromagnetic simulation of the 3D skyrmion string bending effect in a 2D thermal gradient. A mesh size of $128 \times 256 \times 64$ and an elementary cell size of $1 \times 1 \times 1\,\mathrm{nm}^3$ were used. A 2D temperature gradient of $g_\parallel = 9.5$ K/nm and $g_\perp =$

17.5 K/nm was subsequently applied. Other parameters, such as boundary conditions, damping constant, M_S, A, and D etc., are consistent with the former. As shown in Fig. S4 (a), a single skyrmion string moved from the initial straight string state (t=0) to the final bent string state (t=30 ns) after a 2D thermal gradient was applied. The bending of the string can be clearly observed in Fig. S4 (b). As shown in Fig. S4 (c) and (d), the Hall angle Θ_{SkH} of the skyrmion in the front yz-planes is larger than that behind. This is consistent with the results discussed in Fig. 5 in the main text.

S4. MAGNON FRICTION MODEL ANALYSIS

Based on the experimental results, it is able to quantitatively fit the measured data into the magnon friction model.

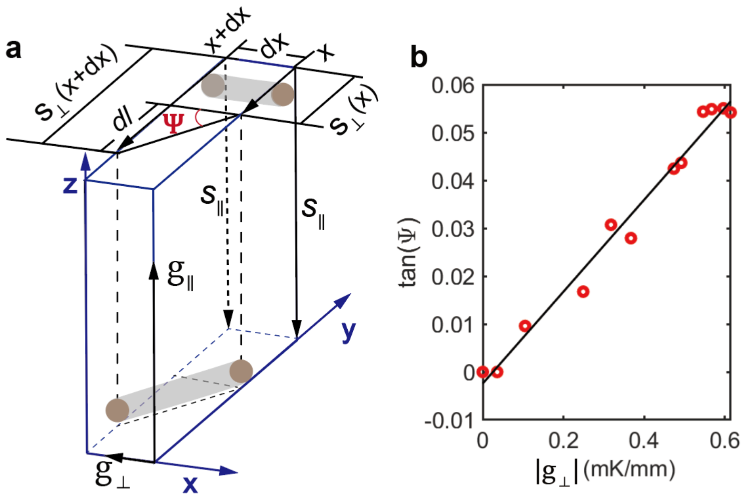

FIG. S5. (a) The geometry of the skyrmion dynamics at two adjacent lateral planes, separated by dx. The sketch shows the skyrmion string's bending angle Ψ to the geometrical properties of the system. (b) Experimentally measured $\tan\Psi$ as a function of $g_\perp$.

As shown in Fig. S5(a), we consider two slices of the yz-planes that are separated by a distance of dx. Note that we employed the identical geometry as that of in the main text. Therefore, the skyrmion Hall angle $\Theta_{\mathrm{SkH}} = v_\perp/v_\parallel$ is x-dependent if the magnon friction is taken into account. By switching on the 2D thermal gradient $g_\parallel$ and $g_\perp$, the skyrmions start to move. Let us assume that our analysis is performed after a reasonable time constant. In this case, the skyrmions in both two slices must have travelled equal distance of $s_\parallel$ along

z-direction, but different $s_\perp$ component long y due to the temperature-dependent skyrmion Hall angle. As sketched in Fig. S5(a), the skyrmion Hall angle for the two slices reads [see Eqn. (2) in our main text]:

$$\tan\Theta_{\mathrm{SkH}}(x) = \frac{s_\perp(x)}{s_\parallel} = \frac{\alpha\mathcal{D} + \eta\bar{T}(x)}{bQ} \quad , \tag{2}$$

$$\tan\Theta_{\mathrm{SkH}}(x+dx) = \frac{s_\perp(x+dx)}{s_\parallel} = \frac{\alpha\mathcal{D} + \eta\bar{T}(x+dx)}{bQ} \quad , \tag{3}$$

where $b = -4\pi M_{\mathrm{S}}/\gamma$. Subtracting the two equations leads to

$$\frac{dl}{s_\parallel} = \frac{\eta g_\perp dx}{bQ} \quad , \tag{4}$$

where $dl = s_\perp(x+dx) - s_\perp(x) = dx\tan\Psi$, as sketched in Fig. S5(a), and it is clear that $\bar{T}(x+dx) - \bar{T}(x) = g_\perp dx$. We therefore obtain the key relation between the observed string rotation angle Ψ and the applied gradient of $g_\perp$:

$$\tan\Psi = \frac{\eta g_\perp s_\parallel}{bQ} \propto \eta g_\perp \quad . \tag{5}$$

Equation (5) essentially establishes the magnon friction model, and can be interpreted as the following: Without the magnon friction bath, one would expect that $\tan\Psi = 0$ at all $g_\perp$ values. If the magnon friction effect would take place, the strings' bending angle of $\tan\Psi$ is a linear function of the applied $g_\perp$, whereas the slope measures the friction's amplitude η. Figure S5(b) shows the measured $\tan\Psi$ value as a function of $g_\perp$. It is thus clear that such a linear profile unambiguously validates the existence of the magnon friction effect.

[1] L. M. Levinson, Investigation of the defect manganese silicide Mn_nSi_{2n-m}, J. Solid State Chem. **6**, 126 (1973).

[2] S. M. Stishov, A. E. Petrova, A. A. Shikov, T. A. Lograsso, E. I. Isaev, B. Johansson, and L. L. Daemen, Lost Heat Capacity and Entropy in the Helical Magnet MnSi, Phys. Rev. Lett. **105**, 236403 (2010).

[3] A. Vansteenkiste, J. Leliaert, M. Dvornik, M. Helsen, F. Garcia-Sanchez, and B. V. Waeyenberge, The design and verification of MuMax3, AIP Adv. **4**, 107133 (2014).

[4] L. Zhao, Z. Wang, X. Zhang, X. Liang, J. Xia, K. Wu, H.-A. Zhou, Y. Dong, G. Yu, K. Wang, X. Liu, Y. Zhou, and W. Jiang, Topology-dependent Brownian gyromotion of a single skyrmion, Phys. Rev. Lett. **125**, 027206 (2020)
